# Prospective, randomized study on the effects of autologous concentrated growth factors in the treatment of cystic lesions of the jaw

**DOI:** 10.1007/s00508-025-02567-x

**Published:** 2025-07-30

**Authors:** Christoph Sacher, Daniel Holzinger, Florian Wagner, Moritz Bechtold, Robert Pillerstorf, Simon Bigus

**Affiliations:** 1 Department of Oral and Maxillofacial Surgery, University Hospital Vienna, Währinger Gürtel 18–20, 1090 Vienna, Austria; 2 Department of Oral and Maxillofacial Surgery, Charité—Universitätsmedizin Berlin, Augustenburger Platz 1, 13353 Berlin, Germany; 3 Tegetthoffstraße 15, 4020 Linz, Austria

**Keywords:** Cystic lesion, Cystectomy, Bone healing, Oral surgery, Wound healing

## Abstract

**Objective:**

The aim of the study was to investigate the efficacy of autologous concentrated growth factors (aCGF) in the treatment and healing of cystic lesions of the jaw.

**Material and methods:**

In this prospective randomized intervention study 138 patients were enrolled, with 68 patients undergoing cystectomy alone and 70 patients undergoing cystectomy with defect filling using autologous concentrated growth factors. Bone healing was volumetrically measured using cone beam computed tomography (CBCT) at 6 and 12 months postoperatively. Clinical follow-ups were conducted 14 days, 1 month, 3 months, 6 months, and 12 months after the treatment.

**Results:**

In both groups, almost complete bone healing occurred, with no significant differences observed between the two groups (*P* =0.484). In the aCGF group there was a trend towards a reduction in wound healing disturbances after 14 days, although this reduction was not statistically significant (*P* =0.071).

**Conclusion:**

The use of aCGF following cystectomy does not show radiologically measurable significantly improved bone healing; however, a tendency towards improved wound healing compared to cystectomy without any filling materials could be observed in the initial healing period.

## Introduction

Cysts are cavities in tissue that can occur in various areas of the body. In the jaws they represent a common clinical issue that requires surgical intervention. Despite various changes, the current treatment options can be attributed to Partsch [1]. who described them over 100 years ago. In small cysts (i.e., up to 1.5 cm^3^ in volume) the main surgical treatment is cystectomy. According to a review by Wakolbinger et al., complete osseous healing can be assumed for these smaller cysts [2].

For cysts exceeding this defect size, there may be an increased risk of coagulum collapse within the defect, leading to both delayed bone healing and an elevated risk of wound healing disturbances. Attempts to stabilize this blood clot have been made using various materials, such as collagen sponges or bone substitute materials [3].

In recent years, a promising therapeutic approach has emerged, namely the application of autologous concentrated growth factors (aCGF). These autologous blood concentrates are increasingly used today to improve both soft tissue and bone healing [4].

The aCGF is a new generation of concentrated platelet products obtained through the centrifugation of patient blood. The growth factors in aCGF play a key role in tissue healing by stimulating cell proliferation, angiogenesis, and collagen synthesis [5, 6]. The use of aCGF in bony defects aims to enhance the regenerative capacity of the jawbone. The clinical application of aCGF, for example in sinus lift procedures, has already been published extensively [7–9]. Compared to the more commonly used platelet-rich fibrinogen (PRF), aCGF contains higher levels of platelets and platelet-derived growth factors due to different centrifugation protocols [10].

The combination of established cystectomy and the use of aCGF provides a potential pathway to accelerate bone healing in the treatment of jaw cysts. The aim of this study was to investigate whether the use of aCGF results in an improvement in healing compared to cystectomy without its application.

## Methods

### Study design

This prospective randomized intervention study was conducted at the University Clinic for Oral and Maxillofacial Surgery at the Vienna General Hospital (Medical University of Vienna).

Patients over 18 years of age, who were admitted to the outpatient ward with a radiological diagnosis of a cystic lesion measuring less than 4 cm on panoramic X‑ray were invited to participate in the study and underwent cone beam computed tomography (CBCT).

Exclusion criteria were the presence of pregnancy or tumorous disease, the (anamnestic) use of bisphosphonates or denosumab and a poor general condition (P3 or higher in the classification of the American Society of Anesthesiologists). After obtaining written consent, a total of 138 patients were initially included, who were then assigned to either the intervention or control group using the randomizer tool of the Medical University of Vienna (https://www.meduniwien.ac.at/randomizer/login). Stratification was performed based on age, gender and smoking status.

### Treatment protocol

The surgical procedures were performed with the patient under local anesthesia or nasotracheal intubation anesthesia, depending on the cyst location and proximity to adjacent anatomical structures such as the inferior alveolar nerve. A cystectomy (Partsch II) was carried out and depending on the assignment to the respective group, the bony defect was either filled with aCGF before wound closure or wound closure alone was performed after blood clot formation.

For the intervention group, peripheral venous blood (20 ml) was directly taken from the patients preoperatively and aCGF was obtained using a centrifuge with a standardized protocol (Medifuge®, Silfradent®, Sofia, Italy; processing time 14 min). No filling material was used in the control group. All patients received preoperative single-shot antibiotic prophylaxis. For patients under local anesthesia, this consisted of oral administration of amoxicillin/clavulanic acid (1 h preoperatively), while for procedures under general anesthesia, intravenous administration of ampicillin/sulbactam 3 g was used preoperatively. In cases of penicillin allergy, moxifloxacin 400 mg was administered orally or intravenously. Postoperative analgesia was achieved with oral paracetamol and, if needed, metamizole.

### Data acquisition, measurements, statistics

The primary outcome parameter was the size of the bony defect, which was determined using CBCT scans. This examination was performed preoperatively as well as at 6 months and 12 months after surgery.

The KaVo 3‑Dimensional eXam device (KaVo Dental Excellence, Biberbach, Germany) was used for the CBCT images, using standardized settings (120 kV, 5 mA, voxel size 0.4 mm, field of view 160 mm × 130 mm).

The cyst or bony defect volumes were evaluated preoperatively as well as residual volumes at 6 and 12 months postoperatively, using radiological software (OsiriX v11.0.3 32 bit, Pixmeo, SARL, Bernex, Switzerland). The intraobserver variability was reduced by standardized volumetric measurements. All measurements were performed at least twice (by one of the authors, CS), and discrepancies of over 5% were subjected to review. Any rupture of the surrounding cortical bone was recorded separately.

The surface of the cyst was manually delineated at a caudocranial slice interval of 1 mm. The target regions in the remaining intermediate slices were completed automatically and reviewed by the examiner, with excessively or inadequately delineated edges being readjusted as necessary.

The volume of the cyst (volume of interest) was then calculated by adding up all delineated areas (cm^3^). A freely rotatable three-dimensional model of the cyst was automatically created by the software (see Fig. 4).

Clinical follow-up examinations were conducted at 14 days, as well as 1, 3, 6, and 12 months postoperatively.

In this context, secondary outcome parameters such as wound healing disturbances, pain, as well as potential sensory deficits and the decayed, missing and filled teeth (DMFT) index were assessed.

The classification of wound healing disturbances was conducted according to the Clavien-Dindo method. Grade 0 indicated no wound healing disorders, grade I required purely conservative wound care with local cleansing and analgesia, grade II necessitated the additional collection of a microbial swab for initially calculated and potentially adjusted antibiotic treatment, and grade III required revision surgery [11].

The collected data were recorded both in a locally stored Excel file and in an analogue case report form for each patient, allowing for data validation in the case of (transmission) errors upon completion. Data analysis was performed using the statistical program “R” (R Core Team, Foundation for Statistical Computing, Vienna, Austria, version 4.3.3).

## Results

Of the 138 patients included 49 patients had to be excluded either because they did not attend the follow-up appointments (46 patients), or because histologically no cyst was present (3 patients). A balance check was conducted and the excluded patients showed no significant differences compared to the patients who were finally included. In the end, 89 patients could be included for further analysis of whom 42 underwent cystectomy alone (47.2%; control group; 18 women, 24 men) and 47 patients underwent cystectomy with insertion of aCGF (52.8%; 20 women, 27 men) and were included in the analysis.

The median age in the control group was 44.0 years (interquartile range, IQR 33.2–53.0 years), and 50.0 years (IQR 43.5–57.0 years; *p* = 0.071) in the aCGF group. Additionally, the groups did not significantly differ in other preoperatively collected criteria (see Table 1).

**Table 1:** Descriptive statistics of the patients included in the study

Parameter		Control group	aCGF	*p*-value
Age (years)	Median (IQR)	44.0 (33.2–53.0)	50.0 (43.5–57.0)	0.071
Sex	Female	18 (42.9)	20 (42.6)	1.000
	Male	24 (57.1)	27 (57.4)	
Smoker	No	25 (59.5)	24 (51.1)	0.523
	Yes	17 (40.5)	23 (48.9)	
Pack-years	Median (IQR)	3.5 (0.0–14.2)	0.0 (0.0–27.7)	0.792
Cortical rupture	No	23 (54.8)	19 (40.4)	0.206
	Yes	19 (45.2)	28 (59.6)	
Tooth extraction	No	10 (23.8)	17 (36.2)	0.253
	Yes	29 (69.0)	28 (59.6)	
OR duration (min)	Median (IQR)	60 (43.8–86.2)	55.0 (41.2–70)	0.477
DMFT index	Median (IQR)	13.5 (10.0–17.5)	18.0 (10.0–24.0)	0.240
Region	Upper jaw	18 (42.9)	17 (36.2)	0.664
	Lower jaw	24 (57.1)	30 (63.8)	

Nominal data are reported in absolute and relative frequencies (in brackets), *p*-value from Fisherʼs exact test; for continuous variables, median and interquartile range (IQR) are shown, *p*-value from Kruskal-Wallis test

The histological analyses predominantly showed radicular cysts, with 24 patients (57.1%) in the control group and 26 patients (55.3%) in the aCGF group. In addition, follicular cysts were observed in 15 patients in the control group (35.7%) and 12 patients in the aCGF group (25.5%). Furthermore, 3 patients in the aCGF group (6.4%) showed nasopalatine cysts, 2 patients (4.3%) exhibited odontogenic keratocysts (aCGF group), and 1 patient (2.1%) presented with a residual cyst (aCGF group). In 3 patients from the control group (7.2%) and 3 patients from the aCGF group (6.4%), no specific cyst diagnosis could be established due to the presence of only inflammatory tissue and, in some cases, no epithelium or other diagnostically relevant features. There was no statistically significant difference in the distribution pattern of the entities (*p* = 0.515), meaning that the groups can be considered comparable.

**Fig. 1:**
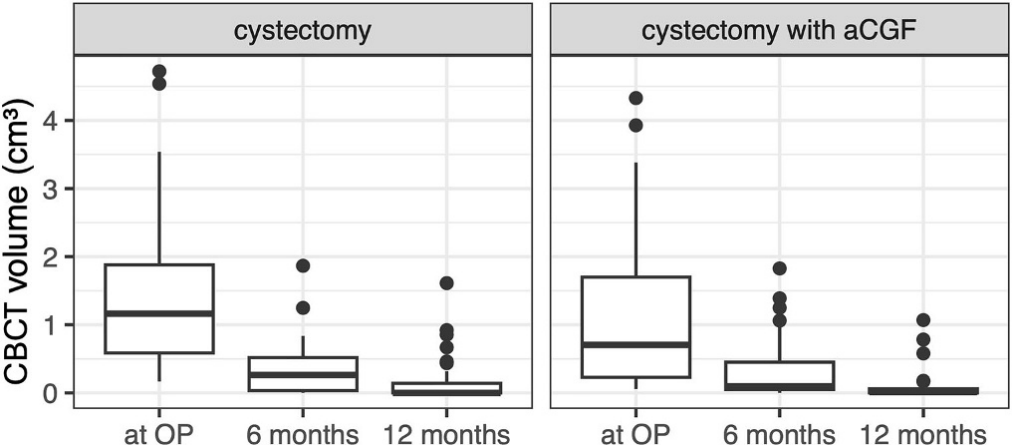
Cyst volumes before surgery, after 6 and after 12 months. Cyst volumes measured preoperatively, after 6 months and after 12 months shown as boxplots, Cystectomy without aCGF (*left*), Cystectomy with aCGF (*right*); the values are given in cm^3^

**Fig. 2:**
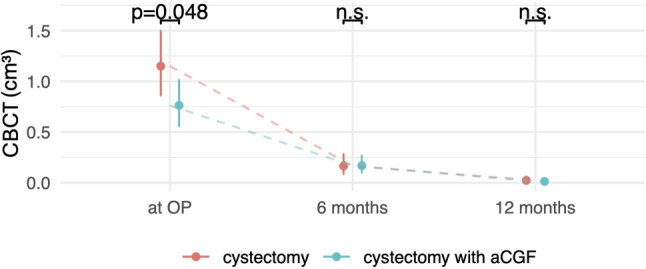
Model predictions CBCT. The cyst volume is shown preoperatively (*OP*), at 6 months and at 12 months. Both groups showed almost complete bone healing, with only a significant difference between the groups preoperatively. *CBCT* cone beam computed tomography

The bone healing and cyst volumes in CBCT are graphically depicted in Figs. 1 and 2. The volume as a target parameter had to be transformed as otherwise the regression assumptions would have been grossly violated. The third root of the volume was approximately normally distributed and was therefore used as an independent variable in the model. A multiple linear regression model was estimated that included an interaction effect between the group indicator and an indicator for the timepoint. In addition, we took sex, age, smoking, cortical rupture and extractions into account, and only found cortical rupture to be a positive significant predictor of CBCT volume (see Table 2). An adjusted R^2^ of 0.497 indicates a good model fit, and regression diagnostics showed no signs of systematic violation of regression assumptions (normality of residuals, linearity, homoscedasticity).

**Table 2:** Regression table: CBCT volume

Predictors	Estimates	Std. error	Std. beta	Standardized std. error	CI	Standardized CI	*p*
(Intercept)	0.93	0.09	0.91	0.16	0.75–1.12	0.59–1.22	**<** **0.001**
CBCT (6 months)	−0.47	0.07	−1.08	0.17	−0.61–−0.32	−1.42–−0.74	**<** **0.001**
CBCT (12 months)	−0.78	0.07	−1.80	0.17	−0.92–−0.64	−2.13–−1.47	**<** **0.001**
Group (cystectomy with aCGF)	−0.18	0.07	−0.41	0.16	−0.31–−0.04	−0.72–−0.10	**0.009**
Age	0.00	0.00	0.05	0.05	−0.00–0.00	−0.05–0.14	0.352
Sex (male)	0.04	0.04	0.10	0.10	−0.04–0.13	−0.09–0.29	0.302
Smoker (Yes)	−0.00	0.04	−0.00	0.10	−0.09–0.08	−0.20–0.19	0.967
Corticalis rupture (Yes)	0.12	0.04	0.28	0.10	0.04–0.20	0.09–0.47	**0.004**
Tooth Extractions (Yes)	−0.03	0.05	−0.08	0.11	−0.12–0.06	−0.28–0.13	0.473
CBCT (6 months) × group (cystectomy with aCGF)	0.11	0.10	0.26	0.23	−0.08–0.31	−0.19–0.71	0.255
CBCT (12 months) × group (cystectomy with aCGF)	0.12	0.10	0.28	0.23	−0.07–0.31	−0.17–0.72	0.221
Observations	232						
R^2^/R^2^ adjusted	0.518/0.497						

Multiple linear regression: The outcome variable is the cube root of the CBCT volume. This value is significantly lower preoperatively in the aCGF group, with no significant difference postoperatively (CBCT6 months and 12 months x group); CBCT (metric), age (metric), sex (dichotomous; 0= male, 1=female), Smoker (dichotomous; 0=no, 1=yes), Corticalis rupture (dichotomous; 0=none; 1 = yes), tooth extractions (dichotomous; 0 = no; 1 = yes); p-values <0,05% are printed in bold.

For the visual representation of the results, a back-transformation to the original scale in cm^3^ has been carried out. Figure 2 shows the predicted CBCT volume (and 95% confidence intervals) at the three timepoints with hypothesis testing for the group comparisons at the timepoints. Preoperatively, a significantly higher defect size (*p* =0.048) was observed in the control group compared to the aCGF group. Almost complete radiological healing of the lesions was found at the last follow-up in both groups. There was no significant difference between the groups. In addition, a random-intercept model with an interaction of the measurement timepoint and group indicator was calculated. The results confirm the findings of the multiple linear regression that the initial CBCT volume is significantly higher in the cystectomy group than in the cystectomy with aCGF group, with no significant difference in CBCT volume at other timepoints (see Table 2).

Wound healing disorders were assessed during the postoperative clinical follow-ups according to the Clavien-Dindo method. These assessments were conducted at the follow-ups after 14 days, 1 month, 3 months and 6 months.

At the first wound check after 14 days, 6 patients in the control group (14.3%) and 1 patient in the aCGF group (2.1%) had a wound healing disorder classified as grade 1 (treated with local cleansing and analgesia), 1 patient from the control group and 2 patients from the aCGF group (2.4% vs. 4.3%) required treatment with antibiotics (grade 2).

**Fig. 3:**
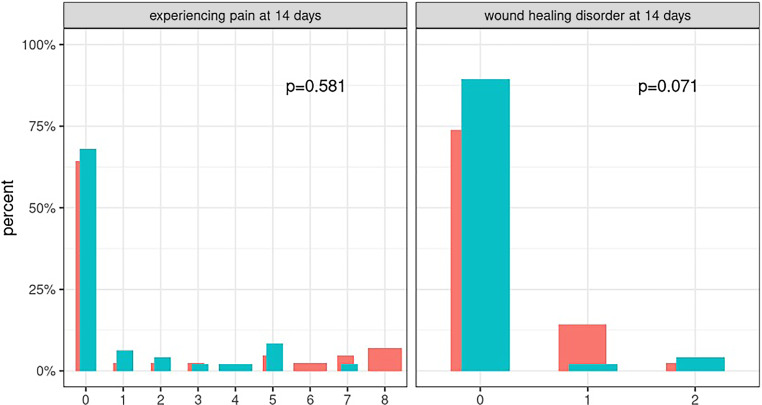
Visual analogue scale (VAS) pain score (*left*) and wound healing disorders (*right*) after 14 days. Postoperative pain measured using VAS, assessment after 14 days, no difference observed between the control group (shown in red) and the aCGF group (shown in turquoise); wound healing disturbances measured according to the Clavien-Dindo classification, indicating a non-significant trend towards fewer wound healing disorders with the use of aCGF. The *p*-value for “experiencing pain” is from the Kruskall-Wallis test, and the *p*-value for “wound healing disorder” is from Fisher’s exact Ttest

In the postoperative healing phase, a clinical trend in favor of the aCGF group was observed, although this reduction was not statistically significant (*P* =0.071, see Fig. 3).

**Fig. 4:**
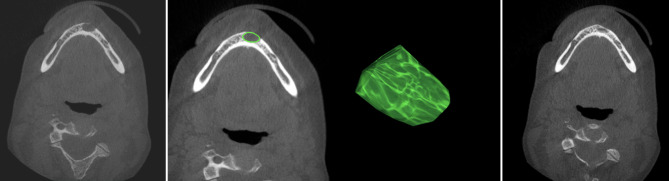
CBCT scans and 3D model of a cyst. CBCT scans: preoperative CBCT of a patient with a suspected radicular cyst (image on the left), one CBCT layer with marked ROI (*in green, left image in the middle*), and a three-dimensional model of the cyst (calculated with OsiriX, right image in the middle). The same patient 6 months postoperatively: the defect has healed almost completely (image on the right).

After 1 month, this trend was no longer observable. In both groups, one patient still required local therapy (grade 1), and in one patient of the control group and two patients of the aCGF group, antibiotic therapy was necessary (grade 2; *p* = 1.0; see Fig. 3).

Postoperative pain was assessed using a VAS scale, where values between 0 and 10 were possible. Here, 0 indicated no pain, and 10 represented the worst imaginable pain. No difference in pain symptoms between the two groups could be demonstrated (*p* =0.581; see Fig. 3). Thus, the use of aCGF did not result in reduced postoperative pain.

Regarding the other potential influencing factors on bone healing, such as age or smoking, no significant differences were observed. A cortical rupture through the bone limiting the lesion was present in 23 patients (54.8%) in the control group and 19 patients (40.4%) in the aCGF group (*p* =0.206), 25 patients in the control group (59.5%) and 24 patients in the aCGF group (51.1%) reported cigarette consumption (*p* =0.523), with no significant difference in the number of pack-years between them. Comparable numbers of root apex resections (*p* =0.815) and extractions (*p* =0.253) were performed in both groups, and there was no significant difference between the groups regarding preoperative dental status (assessed as DMFT index) or the localization of the cysts (see Table 1).

## Discussion

The aim of the present study was to examine potential differences in bone healing of cystic lesions of the jaw after treatment with the conventional cystectomy, without the use of any filling material and cystectomy, followed by filling with aCGF in CBCT scans.

A limitation of this study was a high drop-out rate, which can be explained partly by the low level of pain suffered after surgical treatment. Additionally, some follow-up appointments were impossible due to COVID-19 measures in effect at the time. To exclude potential bias, a balance check was performed comparing excluded patients with those included in the analysis. This test showed no evidence that excluded patients systematically differed from those with completed follow-ups. Another limitation is the assessment of bone healing solely through radiological measurements; no second surgery or biopsy was performed, preventing histological evaluation of bone healing; however, it should be noted that histological evaluations of bone augmentations following the use of aCGF do exist. For example, biopsies after sinus lift surgeries by Sohn et al. are available, showing newly formed bone without inflammatory reactions [7].

In this study, in both groups more men were included. Dentogenic cysts are more commonly diagnosed in men, which may be attributed to their irregular dental check-ups and increased traumatic injuries to the anterior teeth [12, 13].

The histological diagnoses in this study corresponded in frequency to typical distribution patterns. Johnson et al. described approximately 54.6% radicular cysts and 20.6% follicular cysts in a review study [14]. Similar frequency distributions were reported by Rioux-Forker et al., with 52–70% radicular cysts and around 20% follicular cysts [15].

Numerous studies and review articles on bone healing with and without the use of filling materials are now available [3, 12]. The use of aCGF has been investigated in various oral surgical indications such as sinus lift surgeries, guided bone regeneration for bone augmentation, and periodontal surgery [8, 16–19]. In these studies, a predominantly positive influence on bone healing and postoperative symptoms such as swelling and pain has been observed [18]. In the present study, both the control group and the aCGF group showed almost complete radiological healing of the lesions, with no significant difference between the groups; however, a non-significant trend toward fewer wound healing disorders with the use of aCGF was observed. Furthermore, no significant reduction in postoperative pain was achieved with aCGF.

It remains unclear at what size complete healing of defects can be achieved without the use of filling materials [12, 17]. The use of bone substitute material appears to lead to a higher rate of wound healing disorders [20], without any radiologically measurable advantage effect on the bone healing over cystectomy alone, while the use of autologous bone naturally leads to increased postoperative pain and possible complications at the donor site [21].

The duration of bone regeneration depends on the size of the cyst [2, 3], and faster healing would be crucial, especially considering the often desired and necessary dental regeneration (implants) for the patient. Although a positive effect of aCGF on bone healing has been demonstrated in vitro and in vivo [4], this was not the case in our clinical study.

Other potential influencing factors, such as age, gender, smoking or the presence of a rupture of the cortical bone, were investigated. In accordance with our findings, Rubio et al. found no correlation between patient age and bone healing [22].

Studies investigating a possible influence of gender on bony healing usually found better healing in women [12, 20]. In this study, however, no such trend could be observed.

The influence of smoking on wound healing in general is undisputed [23]; however, no effect of smoking on bone healing was found in this study.

The perforation of the cyst through the cortical bone also showed no significant influence on the healing of the lesion; however, care was taken during the surgical interventions to preserve the periosteum in the area of the cortical breakthrough. The preservation of the periosteum and the cortical boundaries was a determinant criterion for proper bone healing in a review study by Buchbender et al. [3, 24].

## Conclusion

This study showed that the use of aCGF tends to have a positive effect on primary wound healing; however, no statistically significant improvements in overall bone healing were demonstrated in radiological measurements. Histological and histomorphometric evaluations and a larger study population would be of particular interest for future studies.

Small cystic lesions of the jaw showed equally good radiological bone healing 6 and 12 months after cystectomy, both with and without defect filling with aCGF, regardless of sex, age, gender and smoking status. Defect filling with aCGF has a positive effect on primary wound healing in the first 2 weeks postoperatively, although no statistical significance was observed.
